# Automatic Sex Determination of Skulls Based on a Statistical Shape Model

**DOI:** 10.1155/2013/251628

**Published:** 2013-11-07

**Authors:** Li Luo, Mengyang Wang, Yun Tian, Fuqing Duan, Zhongke Wu, Mingquan Zhou, Yves Rozenholc

**Affiliations:** ^1^College of Information Science and Technology, Beijing Normal University, Beijing 100875, China; ^2^Laboratoire MAP5, UMR CNRS 8145, University Paris Descartes, 45 rue des Saints-Pères, 75006 Paris, France

## Abstract

Sex determination from skeletons is an important research subject in forensic medicine. Previous skeletal sex assessments are through subjective visual analysis by anthropologists or metric analysis of sexually dimorphic features. In this work, we present an automatic sex determination method for 3D digital skulls, in which a statistical shape model for skulls is constructed, which projects the high-dimensional skull data into a low-dimensional shape space, and Fisher discriminant analysis is used to classify skulls in the shape space. This method combines the advantages of metrical and morphological methods. It is easy to use without professional qualification and tedious manual measurement. With a group of Chinese skulls including 127 males and 81 females, we choose 92 males and 58 females to establish the discriminant model and validate the model with the other skulls. The correct rate is 95.7% and 91.4% for females and males, respectively. Leave-one-out test also shows that the method has a high accuracy.

## 1. Introduction

Sex identification from skeletons is a vital work for a forensic anthropological analysis. Previous studies [1–4] indicate that pelvis is the most reliable indicator of sex assessment, and skull is the second one. However, not all forensic cases provide a complete skeleton due to breakage or postmortem destruction, while the skull can be well preserved in most cases since it is composed of hard tissue. So the skull is the most commonly used skeleton part in forensic anthropological analysis.

Traditional skeletal sex assessments principally rely on visual assessments of sexually dimorphic traits. Rogers [5] achieves an accuracy of 89.1% for a historic skeletal collection by using visual morphological traits. By scoring the visual assessments of five cranial traits (glabella, mental, orbit, nuchal, and mastoid), Walker [6] achieves 90% accuracy for modern American skulls via a quadratic discriminant analysis model incorporating scores. According to Daubert [7] and Mohan [8] criteria, Williams and Rogers [9] assess 21 skull characteristics of 50 white European Americans (25 males and 25 females). For a characteristic, if the intraobserver error is no more than 10% and the accuracy is above 80% when it is used separately to identify the sex, the characteristic is defined as a high quality characteristic. They get six high quality characteristics like eyebrow, orbit, and so forth, and the accuracy reaches 94% by utilizing visual assessments of all these six characteristics. Visual assessments depend heavily on physical anthropologists' understanding of population differences in sexual dimorphism. The visual assessment results reported in the paper [10] show that Krogman and Iscan achieve 92% accuracy using the Todd collection, while Stewart only obtains 77% accuracy using some American black skulls from the Terry collection. Ramsthaler et al. [11] use kappa statistics to quantify the disagreement in sex classification performed by two different observers after visual assessment, and the agreement only reaches 90.8%. Moreover, visual assessment of the morphological traits is likely to be inaccurate when performed by an inexperienced observer due to its great subjectivity. With the progress of the digital imaging technology in medicine, discriminant analysis for skeletal measurements is increasingly used for sex estimation.

Since radiograph can provide architectural and morphological details of the skull, some researchers [12–15] use skull radiograph for sex identification. Hsiao et al. [12] consider the lateral radiographs of 50 male and 50 female adult skulls from Taiwan. They use 18 cephalometric variables, which are derived from some cephalometric points on the lateral radiographs, to build the discriminant function, and claim a coincidence rate of 100%. But they do not mention the generalization ability of the discriminant function, that is, the classification ability for new samples. Veyre-Goulet et al. [15] validate the method of Hsiao by using the lateral radiographic data of 114 Europeans. They find that the result using 8 cephalometric variables is the same as the one using 18 variables, and the coincidence rate is 95.6%. They think that different cephalometric variables can be used for different races. Inoue et al. [14] compute the gradients and distances of 39 measuring points in the lateral contour of the skull and establish the discriminant function through these variables by using 50 female and 50 male skulls from Japan as specimens. 21 other specimens are tested, and the mean correct rate is 86%. They conclude that gradient has higher ability in reflecting gender differences than distances.

Other researchers use some variables measured from 3D skulls to establish discriminant functions. Spradley and Jantz [16] construct multivariate discriminant models using the skull data from the Forensic Anthropology Data Bank. They measure different variables for different races, and the highest identification rate is no more than 90%. Jantz and Ousley [17] issue the computer-aided software called Fordisc for skeleton analysis. This software measures skeleton characteristics by the human-computer interactive way and utilize the data from American Forensic Data Bank to establish the discriminant function. Guyomarc'h and Bruzek [18] compare the sex identification effects of the software for different races such as Thailand and France, and the correct recognition rate is between 52.2% and 77.8%. Ramsthaler et al. [19] test this software by using 98 Caucasian German skulls, and the correct rate is 86%. These studies show that the craniofacial morphology has great diversities among different races. For Chinese, Li [20] manually extracts the mid-sagittal frontal arc on dried skulls from northeast China, and the Fourier coefficients of the arc are used for multivariate stepwise discriminant analysis. The recognition rate for 31 test skulls is 84.21% and 83.33% for males and females, respectively. Shui [21] measures 14 metric variables and utilizes 94 skulls from north China to establish stepwise fisher discriminant functions. The recognition rate for 39 test skulls reaches 87%.

Discriminant analysis for skeletal measurements requires a high measurement precision. However, the accurate measurement of the skull is quite difficult. Williams and Rogers [9] show that, for most measure variables, usually the measurement error among different observers is above 10%. Moreover, the skull size changes with varying ages. In this work, we propose an automatic sex determination method for 3D digital skulls. A statistical shape model is established to describe statistical features of the skull morphology, and Fisher discriminant analysis of the shape parameters is used to classify skulls. The advantages of this method is as follows: Firstly, it needs no professional qualification and tedious manual measurement; Secondly, it is less influenced by the variation of the skull size; Finally, it can get a high recognition rate.

## 2. Materials and Methods

### 2.1. Materials

This study has been approved by the Institutional Review Board (IRB) of Image Center for Brain Research, National Key Laboratory of Cognitive Neuroscience and Learning, Beijing Normal University. It is carried out on a database of 208 whole skull CT scans on voluntary persons that mostly come from Han ethnic group in North of China, age 19–75 years for females and 21–67 years for males. There are 81 females and 127 males. The CT images were obtained by a clinical multislice CT scanner system (Siemens Sensation 16) in the Xianyang hospital located in western China. The images of each subject are restored in DICOM format with a size of approximately 512 × 512 × 250. Each 3D skull surface is extracted from the CT images and represented as a triangle mesh including about 150,000 vertices. All the skulls are substantially complete; that is, each skull contains all the bones from calvarias to jaw and has the full mouth of teeth.

**Figure 1:**
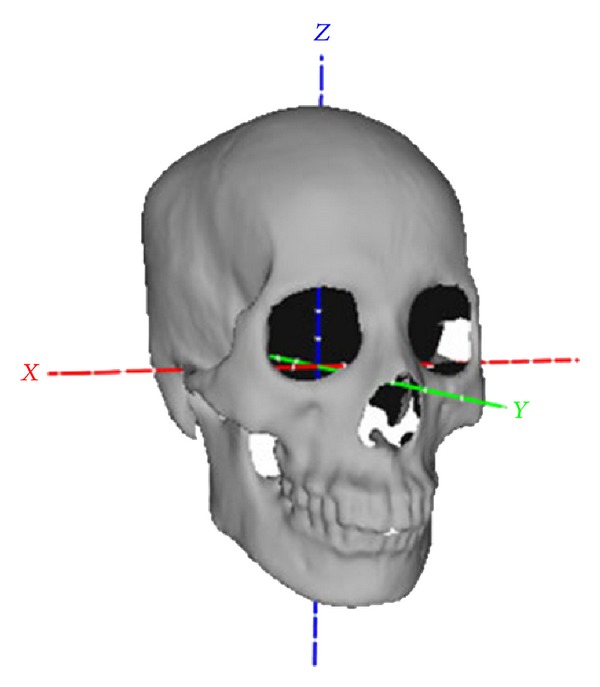
One skull in the uniform coordinate system.

To eliminate the inconsistence in position, pose, and scale caused by data acquirement, all the samples are transformed into a uniform coordinate system. The uniform coordinate system is determined by four skull landmarks, left porion, right porion, left (or right) orbitale, and glabella (denoted as *L*_*p*_, *R*_*p*_, *L*_*o*_, *G*). From three points, *L*_*p*_, *R*_*p*_, *L*_*o*_, the Frankfurt plane is determined [22]. The coordinate origin (denotes as *O*) is the intersection point of the line *L*_*p*_ *R*_*p*_ and the plane that contains point *G* and orthogonally intersects with line *L*_*p*_ *R*_*p*_. We take the line *OR*_*p*_ as *x*-axis. The *z*-axis is the line through the point *O* and with the direction being the normal of the Frankfurt plane. Then *y*-axis is obtained by the cross product of *z*- and *x*-axis. Once the uniform coordinate system is defined, all the prototypic skulls are transformed into it. Finally, the scale of all the samples is standardized by setting the distance between *L*_*p*_ and *R*_*p*_ to unit; that is, each vertex (*x*, *y*, *z*) of the skull is scaled by (*x*/|*L*_*p*_ − *R*_*p*_|, *y*/|*L*_*p*_ − *R*_*p*_|, *z*/|*L*_*p*_ − *R*_*p*_|). One skull in the uniform coordinate system is shown in Figure 1.

### 2.2. Statistical Shape Model Construction

**Figure 2:**
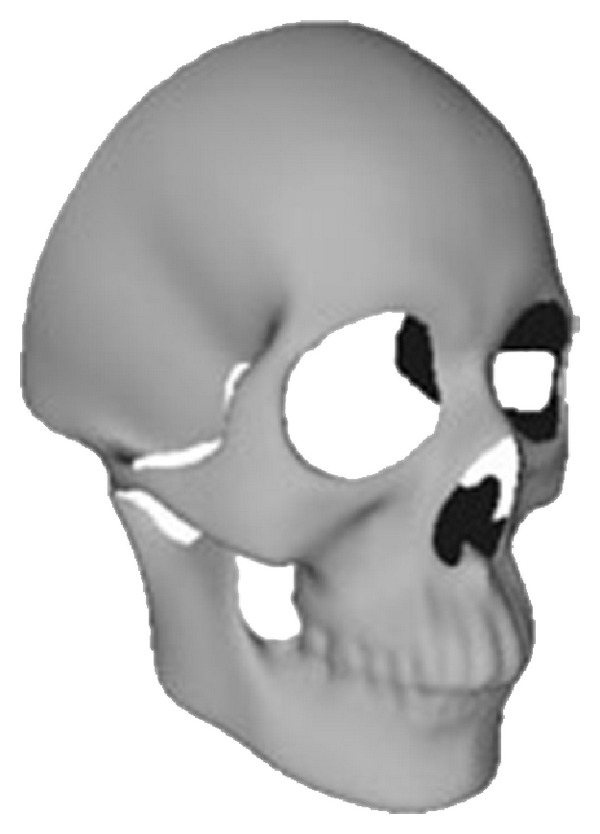
The back part of the reference skull is cut away.

Statistical shape model is a widely used technique in medical image analysis. It can efficiently describe the shape variance and ensure that only statistically likely shapes are represented. Principal Component Analysis (PCA) [23] is a powerful tool to build statistical shape models, and it finds the major and minor modes of shape variation across the training data. In order to build the statistical model, a dense point correspondence has to be established across the training set, that is, building a point-to-point correspondence for all training samples according to human anatomy characteristics. There are many nonrigid registration methods [24–26] for dense mesh or point cloud objects, and here we adopt the method presented by Hu et al. [25]. As in [25], the back part of the reference skull is cut away (as shown in Figure 2) considering that there are too many vertices in the whole skull, and the sexually dimorphic traits are mainly on the front part of skulls. So all of the aligned training skulls only keep the front part corresponding to the reference.

By concatenating coordinates of all the vertices, a skull can be represented as a high dimension vector. Thus we construct a dataset of skulls {**S**_*i*_ = (*x*_*i*1_^*S*^, *y*_*i*1_^*S*^, *z*_*i*1_^*S*^,…, *x*_*im*_^*S*^_  _, *y*_*im*_^*S*^, *z*_*im*_^*S*^_  _)^*T*^ | *i* = 1,2,…, *N*}, where each coordinate index labels corresponding points across the training set.

From the skull dataset, the mean skull data $\overset{-}{S}$ and covariance matrix Σ of the mean normalized skulls are calculated. PCA essentially transforms the mean normalized shape data into a subspace spanned by the orthogonal unit eigenvectors **U**_*k*_,  *k* = 1,2,…, *N* − 1, of the covariance matrix in descending order according to their associated eigenvalues *λ*_*k*_, which represent variation modes of the data. Then the skull statistical shape model is constructed as in the following parameterized model:

(1) $$\begin{array}{c} S\left(a\right)=\bar{S}+\sum_{k=1}^{M}a_{k}U_{k}, \end{array}$$

where the number *M* is the mode number, usually determined by a variance contribution rate calculated from the cumulative eigenvalues, and the combination coefficient **a** = (*a*_1_,*a*_2_,…,*a*_*M*_)^*T*^ is the model parameter. Apparently this statistical shape model assumes that the shape vectors **S** obey a normal distribution with mean $\overset{-}{S}$ and covariance matrix Σ, so the parameter **a** for a plausible skull meets a normal distribution with zero mean and covariance matrix diag⁡(*λ*_1_, *λ*_2_,…, *λ*_*M*_).

### 2.3. Statistical Shape Model Matching

Model matching is to determine the model parameter for a given skull. If the skull data **S**_0_ is aligned, the model parameters can be determined by PCA transform. According to the statistical models (1), let **P**_*s*_ = [**U**_1_, **U**_2_,…, **U**_*M*_] denote the PCA transform matrix for skulls, and the model parameter can be determined as follows:

(2) $$\begin{array}{c} a=P_{s}^{T}\left(S_{0}-\overset{-}{S}\right). \end{array}$$

**Figure 3:**
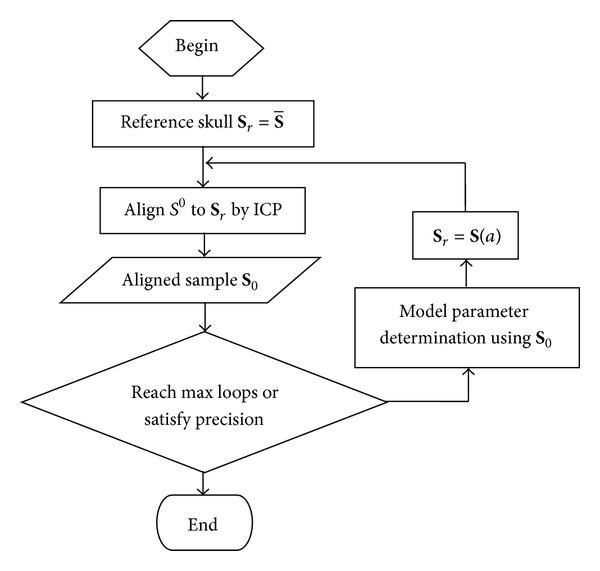
Model matching procedure.

So in fact the model matching is a procedure of the skull registration. Given an unknown prototypic skull data *S* ^0^, it is firstly transformed into the uniform coordinate system as Section 2.1 describes.  Figure 3 shows a statistical shape model based registration algorithm. As shown in Figure 3, a dynamic reference, denoted as **S**_*r*_, is updated by the statistical shape model in each loop, whose model parameter is determined by the PCA transform of the corresponding aligned sample **S**_0_ of last iteration. The initial reference is selected as the mean shape, and an ICP algorithm [27] is used to align a target to the reference. Apparently, the dynamic reference will be closer and closer to the target skull along with the iterating, so the iteration will converge. When the aligned sample does not change, the iteration stops.

### 2.4. Fisher Discriminant Analysis in the Shape Parameter Space

Fisher discriminant analysis is increasingly used for skull sex determination. It projects samples from a high-dimensional feature space into one axis called Fisher vector, in which optimal linear classification can be achieved. Different from previous methods, we perform Fisher discriminant analysis not for skull measurements, but for shape parameters of skulls.

According to the statistical shape model (1), a skull is represented as a feature vector **a** = (*a*_1_,*a*_2_,…,*a*_*M*_)^*T*^. Let **m**_*i*_, *n* ^*i*^, *i* = 1,2, denote the mean and the number of the training samples of the *i*th class, and let **X**_*j*_^*i*^ be the feature vector of the *j*th sample of the *i*th class. Then within-class scatter matrix and between-class scatter matrix can be defined as follows:

(3) $$\begin{array}{c} S_{w}=\sum_{i=1}^{2}\left(\sum_{j=1}^{n^{i}}\left(X_{j}^{i}-m_{i}\right){\left(X_{j}^{i}-m_{i}\right)}^{T}\right),\\ S_{b}=\left(m_{1}-m_{2}\right){\left(m_{1}-m_{2}\right)}^{T}. \end{array}$$

The Fisher criterion is that the samples of the same class are aggregated in the Fisher vector space, while the samples of different class are separated as much as possible. So the objective function is defined as follows:

(4) $$\begin{array}{c} J_{F}\left(w\right)=\frac{w^{T}S_{b}w}{w^{T}S_{w}w}. \end{array}$$

By Lagrange Multiplier, the Fisher vector to this maximization problem is as follows:

(5) $$\begin{array}{c} w=S_{w}^{-1}\left(m_{1}-m_{2}\right). \end{array}$$

Then each skull feature vector **a** can be projected to the Fisher vector, in which two classes of samples can be well separated:

(6) $$\begin{array}{c} t=W^{T}a. \end{array}$$

Finally, a threshold is selected by using some prior knowledge, for example,

(7) $$\begin{array}{c} t_{0}=W^{T}\frac{\left(m_{1}+m_{2}\right)}{2}. \end{array}$$

Given an unknown skull, the procedure of the sex determination is as follows.

*Step  1*. Perform the statistical shape model matching to determine the shape parameter as Figure 3 describes.

*Step  2*. Project the shape parameter to the Fisher vector, and get the projection value *t*.

*Step  3*. If the value is larger than the threshold, the skull gender is male. Otherwise, it is female.

## 3. Results

**Figure 4:**
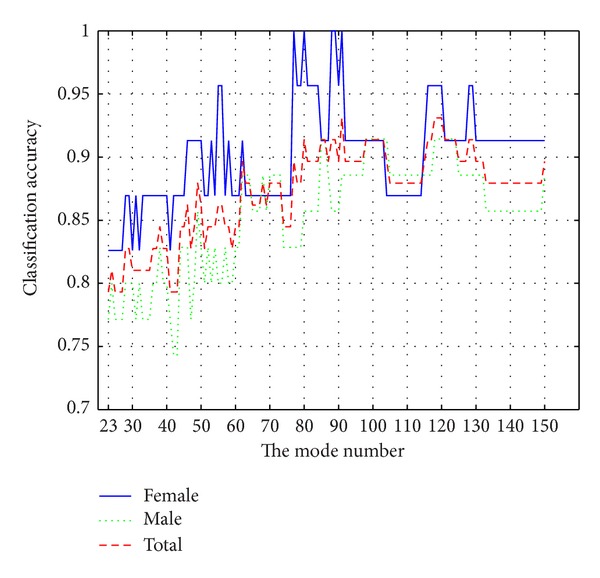
Variation of the correct classification rate of the test samples with the mode number.

The used data are the 208 whole skull CT scans described in Section 2. We chose about three-fourths of the 208 skulls according to the age distribution as training samples, including 92 males and 58 females, and other skulls as test samples. In order to determine an appropriate mode number (*M* in (1)) to build the statistical shape model, we use the trial and error technique and analyze the variation of the classification accuracy of the test samples with varying the mode number by experiments. It is well known that, in PCA, generally only some eigenvectors corresponding to large eigenvalues of the covariance matrix represent modes of variation of the data, while others represent noises. We vary the mode number from 23 to 150, corresponding to variance contribution rate from 96% to 100%, and the correct classification rates for test samples are shown in Figure 4, while the rate of training samples is 100% when the mode number is larger than 80. From Figure 4 we can see that the correct classification rate of females is always higher than that of males, both of the correct rates for females and males are above 86% when the mode number is greater than 80, and the total correct rate for all test samples reaches maximum with the mode number being 91, 118, 119, and 120. Since the correct rate is stable with the mode number varying from 118 to 120, we use 119 modes to build the statistical shape model.

**Figure 5:**
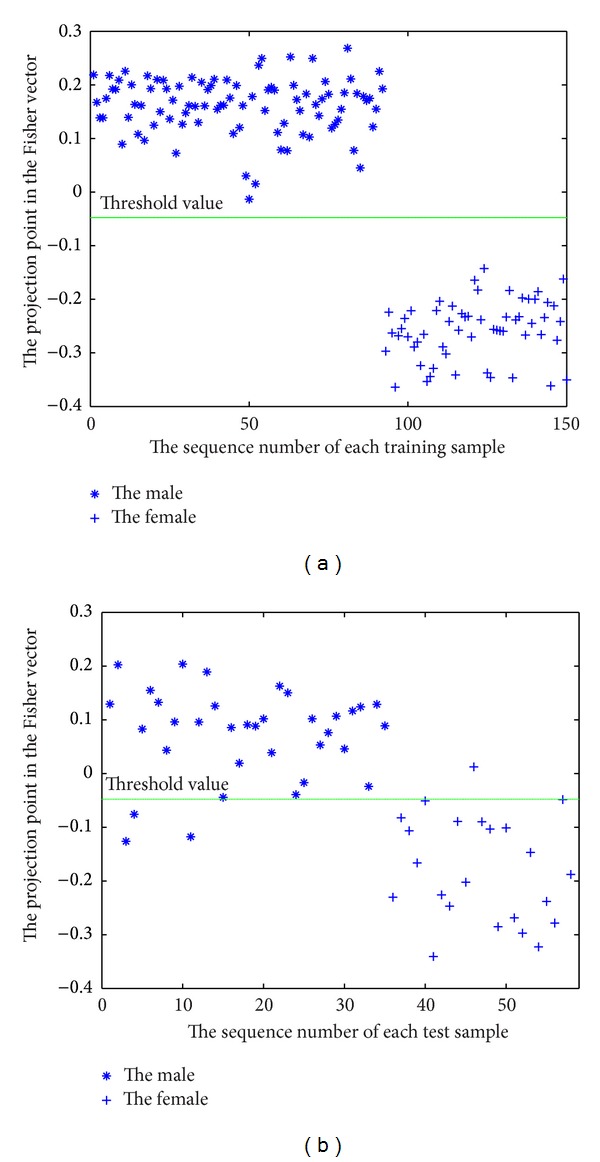
Classification of training samples (a) and test samples (b).

Figure 5(a) shows the classification of the training samples with the mode number being 119, and the classification accuracy of the training samples is 100%.  Figure 5(b) shows the classification of the test samples. One female and 3 males are misclassified, and the correct rate is 95.7% and 91.4% for females and males, respectively. We also classify the whole 208 samples using leave-one-out strategy. That is, repeatedly 207 samples are chosen as training samples, and the residual one is used as test sample. The mode number is still 119, which maybe is not the optimal selection. The correct classification rate is 91.3% and 90.1% for males and females, respectively. Compared with the accuracy of 87% by Shui [21], which is a metric analysis method and uses a subset of our 208 skulls to establish the discriminant functions, this method highly improves the accuracy.

## 4. Discussion

Traditional morphological methods depend heavily on physical anthropologists' subjective understanding of population differences in sexual dimorphism. Different observers usually have a significant difference when performing the visual assessment of the morphological traits, especially for those inexperienced observers. On the other hand, discriminant analysis for skeletal measurements depends less on the examiner's professional qualification and experience, but it requires a high measurement precision, which is not easy to realize. Majority of scientists contend that the combination of metrical and morphological methods is the best way [28]. In this work, we use 3D dense point cloud data to build the statistical shape model, and in a sense, the model parameters metrically describe the skull morphology. So the method combines the advantages of metrical and morphological methods. Different from previous methods, what this method measures is not distance- or volume-related variables, but global shape variations.

Previous studies [18] show that the performance of sex determination outside the reference population group for which the discriminant function has been developed is poor. We do not know whether this method also has this problem due to lack of skull samples of another population, but there indeed exists the problem of selection of training samples. The more complete the training samples are, the better the performance is. That is, the training samples should depict shape variations as much as possible. We think that only if other populations have similar shape variations, the discriminant model we build is applicable. That just is the reason that the correct classification rate of females is uniformly higher than that of males, as shown in Figure 4. When the statistical shape model and the discriminant function have been established, sex determination of an unknown skull becomes easy to perform, and needs no professional qualification and tedious manual measurement. Moreover, although we use CT scans to construct 3D point cloud model of the skull in this work, the statistical shape model we build also can deal with 3D models constructed in any way such as laser scan 3D camera.
